# Evaluation of the inflammatory and osteogenic response induced by titanium particles released during implantoplasty of dental implants

**DOI:** 10.1038/s41598-022-20100-2

**Published:** 2022-09-22

**Authors:** Jorge Toledano-Serrabona, Begoña M. Bosch, Leire Díez-Tercero, F. Javier Gil, Octavi Camps-Font, Eduard Valmaseda-Castellón, Cosme Gay-Escoda, Mª Ángeles Sánchez-Garcés

**Affiliations:** 1 Department of Oral Surgery and Implantology, Faculty of Medicine and Health Sciences, University of Barcelona, Barcelona, Spain; 2 Bellvitge Biomedical Research Institute (IDIBELL), Barcelona, Spain; 3 Bioengineering Institute of Technology, International University of Catalonia, Sant Cugat del Vallès, Spain; 4 Faculty of Dentistry, International University of Catalonia, Sant Cugat del Vallès, Spain

**Keywords:** Peri-implantitis, Dental implants

## Abstract

Implantoplasty is a mechanical decontamination technique that consists of removing the threads and polishing and smoothing the dental implant surface. During implantoplasty there is a large release of titanium metal particles that might provoke a proinflammatory response and reduce the viability of osteogenic cells. We analyze the inflammatory and osteogenic response induced by Ti6Al4V particles released during implantoplasty and by as-received commercially pure Ti particles. Macrophages stimulated with metal particles obtained by implantoplasty and with as-received Ti particles showed an increased proinflammatory expression of TNF-α and a decreased expression of TGF-β and CD206. Regarding cytokine release, there was an increase in IL-1β, while IL-10 decreased. The osteogenic response of Ti6Al4V extracts showed a significant decrease in Runx2 and OC expression compared to the controls and commercially pure Ti extracts. There were no relevant changes in ALP activity. Thus, implantoplasty releases metal particles that seems to induce a pro-inflammatory response and reduce the expression of osteogenic markers.

## Introduction

Peri-implantitis is defined as a pathological condition caused by bacterial biofilms and characterized by inflammatory changes in the peri-implant mucosa, with progressive bone loss around an osseointegrated dental implant^1^. This biological complication can jeopardize the implant-prosthetic restoration if left untreated. The treatment of peri-implant diseases includes the decontamination of the implant surface by means of mechanical, chemical or electrolytic techniques^2–4^. It must be pointed out that the literature does not clearly indicate superiority of one specific decontamination protocol over the others^4^. In fact, most of these decontamination techniques are not able to completely remove the biofilm from the dental implant surface, probably due to the macro and micro-design of the implant and the defect configuration^5–9^. Clinical studies have suggested that the resolution of the peri-implant disease may be influenced by other factors than the method of surface decontamination^10–12^.

Several studies have confirmed the association between peri-implant diseases and bacterial biofilm^1,13,14^. Nevertheless, other factors might cause inflammation and marginal bone loss around dental implants, as suggested by the 2017 World Workshop on the classification of peri-implant diseases^1^. Among these factors, particles of titanium (Ti) and other metals seem to favour bone loss and inflammation of the peri-implant mucosa^15–18^. Most implants are made of commercially pure titanium or alloys with other metals, and accumulation of Ti in peri-implant tissues has been associated with peri-implantitis^19^. Besides, Ti debris promotes DNA damage in oral epithelial cells through activation of the molecular markers CHK2 and BRCA1^20^.

Macrophages are one of the principal elements in the inflammatory immune response and play an important role in bone homeostasis^21,22^. As a result of the stimuli that these cells are exposed to, macrophages are able to polarize into M1 (proinflammatory phenotype) or M2 (anti-inflammatory phenotype)^23^. In response to particulate foreign body material (i.e., wear metal particles) M1 macrophages can express different pro-inflammatory cytokines, chemokines and growth factors that favours osteoclastogenesis^24,25^.

It has been suggested that metal particles may induce an aseptic bone loss around the dental implants^16,26^. These metal particles can be released into the peri-implant tissues through different mechanisms, including implant insertion, corrosion, friction, or surface decontamination methods of the dental implant, such as implantoplasty (IP). In addition, fretting corrosion can also stimulate the release of metal ions and particles into peri-implant tissues in the absence of bacteria^27–29^. Several in vitro cell-based assays in cultured human macrophages reported that Ti particles induce the secretion of pro-inflammatory cytokines^17,30^. However, the immunological characteristics of metal particles released during IP remain unclear. Such debris has shown physicochemical properties characteristic of implant polishing and has been associated to lower corrosion resistance^31,32^.

Thus, the main objective of this paper was to evaluate the inflammatory response induced by Ti metal particles released during IP based on macrophage cell culture (THP-1). The secondary objective was to evaluate the osteogenic response triggered by such metal debris based on human bone marrow mesenchymal stem cell (BM-MSC) culture.

## Results

### Analysis of cytotoxicity in macrophage cell culture

**Figure 1:**
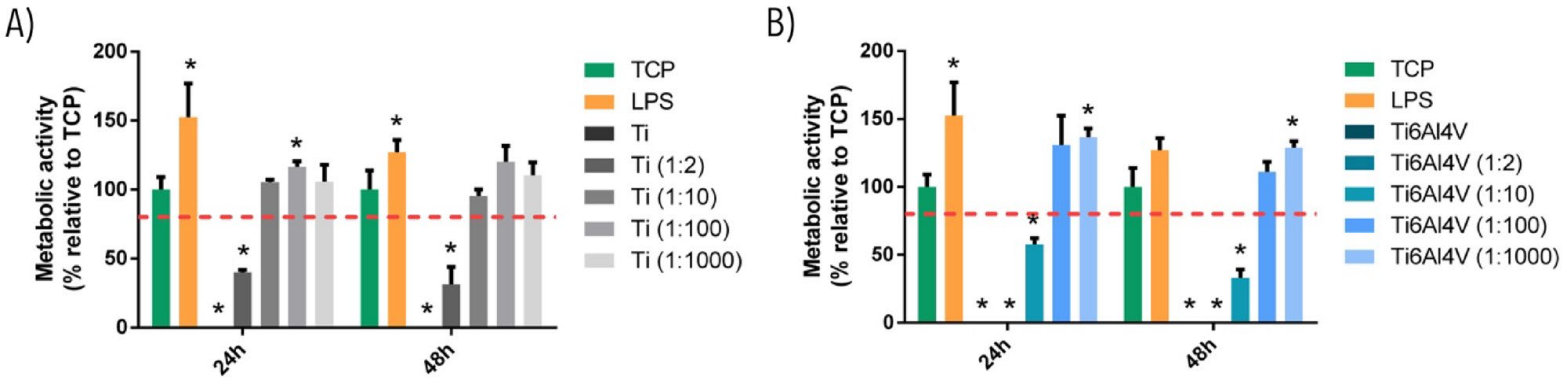
Effect of Ti (**A**) and Ti6Al4V (**B**) particles upon THP-1 cell metabolic activity after 24 h and 48 h culture. Metabolic activity results were represented as percentage relative to an unstimulated control (TCP) and compared with the TCP of each day. Values < 80% metabolic activity (red line) which were significantly different (*p* < 0.05) from TCP were considered cytotoxic. Statistically significant differences (*p* < 0.05) are represented with *.

As shown in Fig. 1, cells cultured with the undiluted extracts of Ti6Al4V and Ti particles and their 1:2 dilution were cytotoxic at 24 and 48 h. In addition, extracts of Ti6Al4V particles were likewise cytotoxic at 1:10 dilution at both 24 and 48 h. Hence, the inflammatory response was assayed with the dilution considered to be non-cytotoxic (1:100).

### Macrophage gene expression analysis

**Figure 2:**
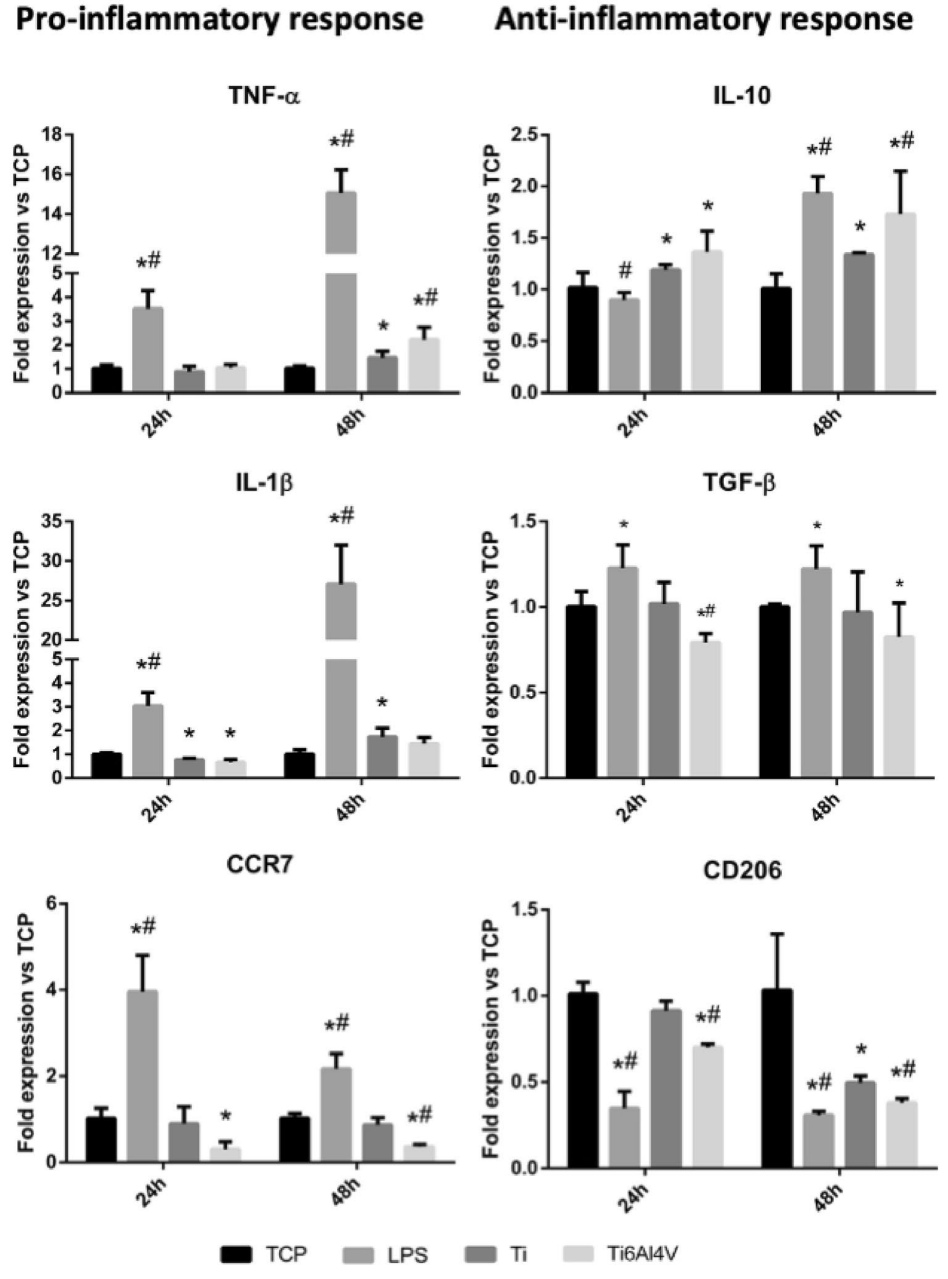
Effect of stimulation with Ti and Ti6Al4V alloy extracts upon gene expression of proinflammatory (TNF-α, IL-1β and CCR7) and anti-inflammatory markers (IL-10, TGF-β and CD206) in the THP-1 macrophage cell line. Statistically significant differences (*p* < 0.05) are represented with * when comparison was made versus TCP and with the symbol # when comparison was made versus Ti.

Lipopolysaccharide (LPS) stimulation produced a statistically significant increase (*p* < 0.05) in the expression of the proinflammatory markers (TNF-α, IL-1β and CCR7) compared to TCP, as well as a decrease in the expression of CD206, which is an anti-inflammatory marker (Fig. 2).

When macrophages were stimulated with the Ti extracts, a significant increase (*p* < 0.05) in TNF-α expression was observed at 48 h of stimulation, while IL-1β expression decreased at 24 h and increased again at 48 h. In addition, at 48 h there was a significant increase in IL-10 expression, as well as a decrease in CD206 expression. On the other hand, macrophages stimulated with Ti6Al4V extracts exhibited an increase in TNF-α expression at 48 h, while IL-1β expression decreased at 24 h, but without differences versus TCP at 48 h. The Ti6Al4V extracts induced a decrease in the expression of the proinflammatory surface marker CCR7.

Lastly, in the Ti6Al4V extracts there was an increase in the expression of TNF-α and IL-10, as well as a decrease in the expression of the CCR7 and CD206 markers at 48 h compared to commercially pure Ti (*p* < 0.05).

### Macrophage cytokine release analysis

**Figure 3:**
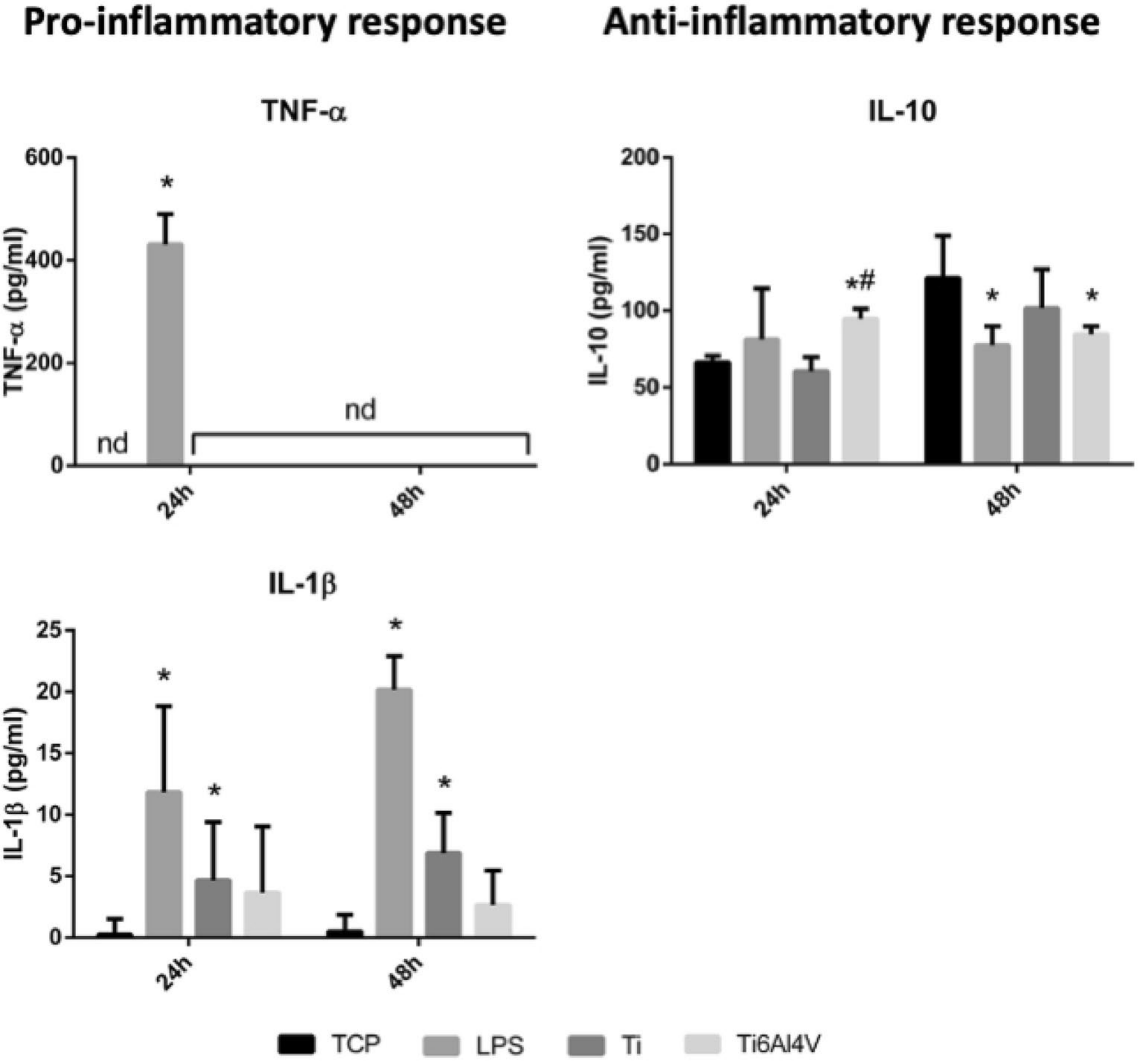
Effect of stimulation with Ti and Ti6Al4V alloy extracts upon the release of proinflammatory (TNF-α and IL-1β) and anti-inflammatory cytokines (IL-10) in the THP-1 macrophage cell line. Statistically significant differences (*p* < 0.05) are represented with * when comparison was made versus TCP and with the symbol # when comparison was made versus Ti.

Only LPS (positive control of inflammation) was able to induce the release of the proinflammatory cytokine TNF-α at 24 h of stimulation. On the other hand, the levels of the proinflammatory cytokine IL-1β were significantly higher (*p* < 0.05) in comparison with TCP in the presence of LPS and Ti extracts, but not with Ti6Al4V (Fig. 3).

At 24 h of stimulation, a significant increase was observed in the expression of the anti-inflammatory cytokine IL-10 in the presence of Ti6Al4V extracts (*p* < 0.05), but we detected a decrease in IL-10 at 48 h. Regarding the Ti extracts, no statistically significant differences were observed with respect to TCP (Fig. 3).

### Analysis of cytotoxicity in human bone marrow-derived mesenchymal stem cells (BM-MSCs) culture

**Figure 4:**
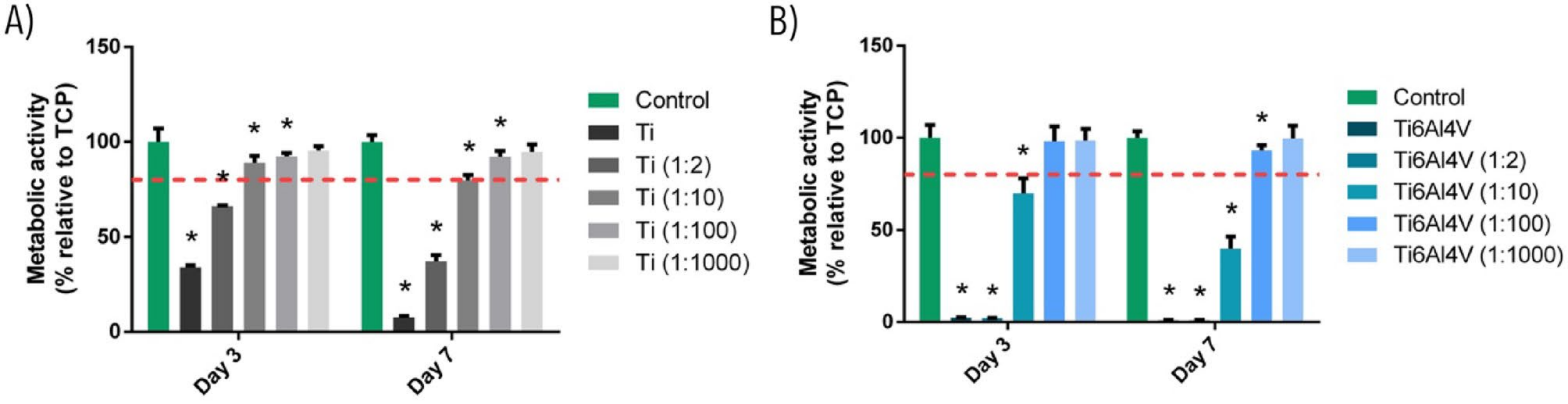
Effect of Ti (**A**) and Ti6Al4V (**B**) particles upon the metabolic activity of human bone marrow-derived mesenchymal stem cells (BM-MSCs) cultured for 3 and 7 days. Metabolic activity results were represented as percentage relative to an unstimulated control (TCP) and compared with the TCP of each day. Values < 80% metabolic activity (red line) which were significantly different (*p* < 0.05) from TCP were considered cytotoxic. Statistically significant differences (*p* < 0.05) are represented with *.

As shown in Fig. 4, undiluted extracts of Ti6Al4V and Ti particles and their 1:2 dilution were cytotoxic for BM-MSCs at 3 and 7 days. In addition, extracts of the alloy particles were likewise cytotoxic at 1:10 dilution at both 3 and 7 days. Although a statistically significant decrease in metabolic activity was observed for Ti extracts at 1:10 and 1:100 dilution and Ti6Al4V extracts at 1:100 dilution, these concentrations were considered to be non-cytotoxic, because they were above the cytotoxicity threshold. Therefore, for the following experiments corresponding to the analysis of osteogenic response, the 1:100 dilution was used in both types of extracts.

### BM-MSCs gene expression analysis

**Figure 5:**
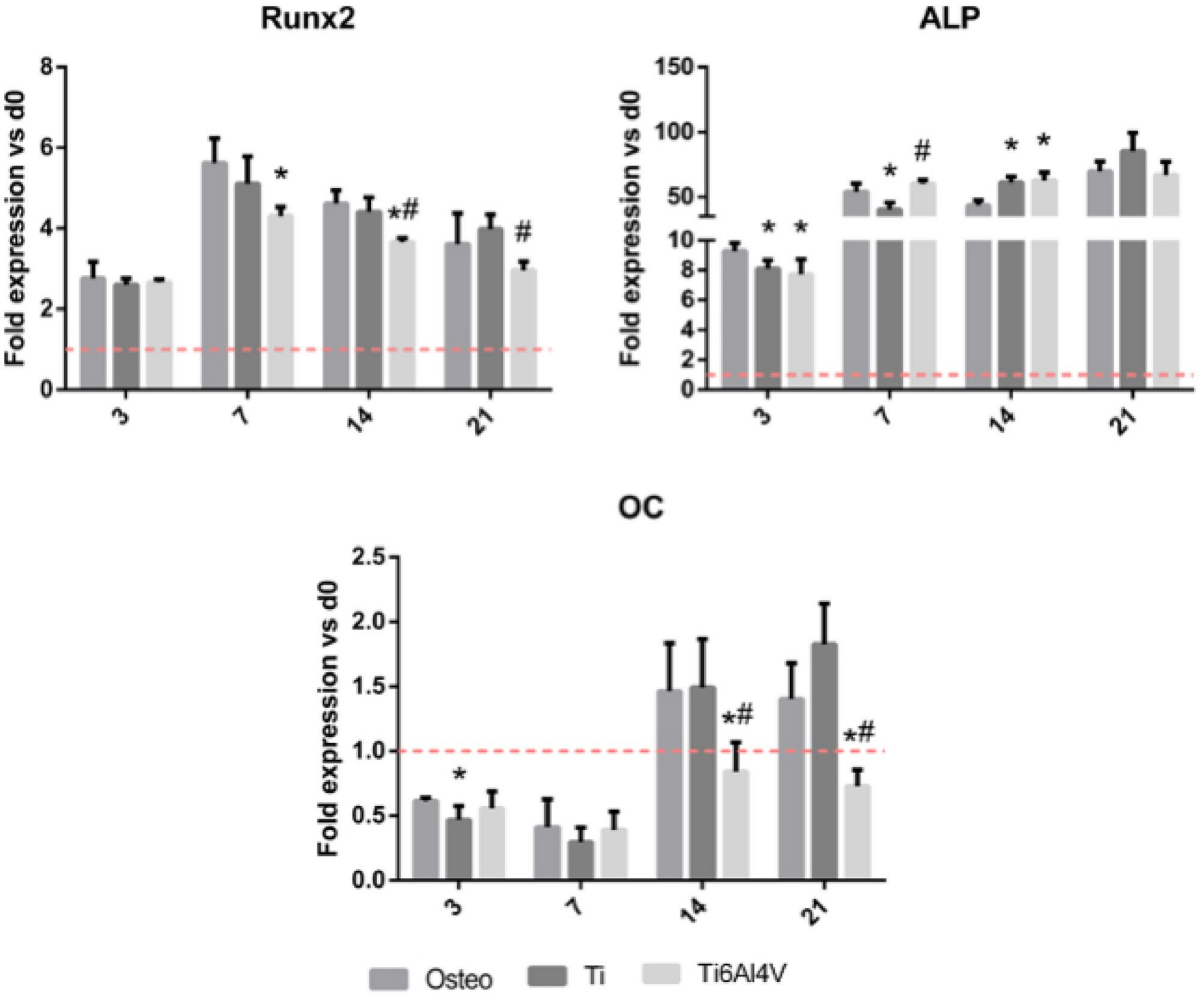
Effect of stimulation with Ti and Ti6Al4V alloy extracts upon gene expression of osteogenic markers Runx2, ALP and OC in cultured human bone marrow-derived mesenchymal stem cells (BM-MSCs). Statistically significant differences (*p* < 0.05) are represented with * when comparison was made versus osteogenic medium (Osteo) and with the symbol # when comparison was made versus Ti.

The Ti6Al4V extracts produced a significant decrease (*p* < 0.05) in Runx2 expression at days 7, 14 and 21 compared to the controls, and at days 14 and 21 compared to cells stimulated with Ti extract (Fig. 5). Additionally, stimulation with Ti6Al4V extracts induced a decrease in the expression of the OC marker at days 14 and 21 compared to the control osteogenic medium and to the Ti extracts. On the other hand, stimulation with the Ti extracts did not produce significant differences versus the controls in terms of Runx2 expression.

Regarding ALP expression, the use of extracts produced a significant decrease at day 3, while at day 14 this reduction could only be seen in Ti extracts. At day 14, expression in the presence of both extracts significantly (*p* < 0.05) exceeded the controls.

### Alkaline phosphatase (ALP) activity assay

**Figure 6:**
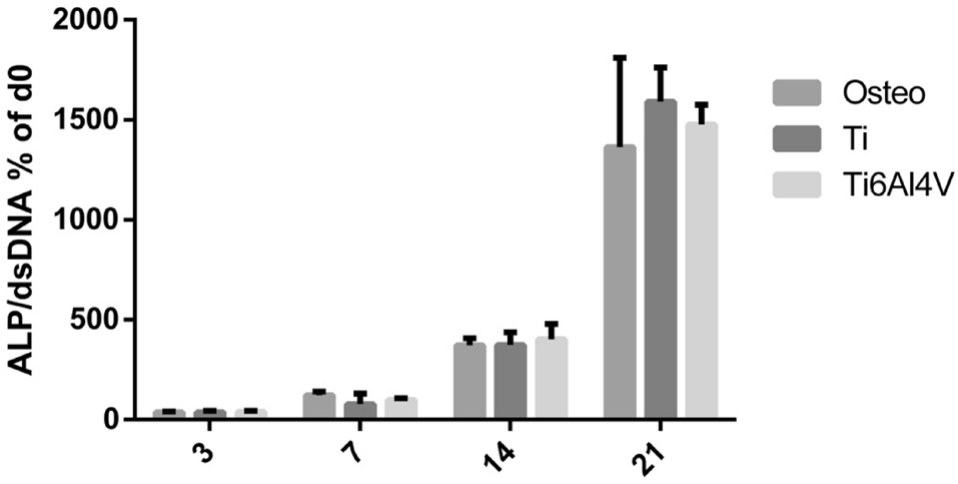
Effect of stimulation with Ti and Ti6Al4V alloy extracts upon ALP activity in cultured human bone marrow-derived mesenchymal stem cells (BM-MSCs). Statistically significant differences (*p* < 0.05) are represented with * when comparison was made versus TCP and with the symbol # when comparison was made versus Ti.

ALP activity was not significantly different when compared to the control osteogenic medium. The Ti6Al4V and Ti extracts showed no statistically significant differences, which indicates that these extracts had no detectable influence upon ALP protein expression and activity (Fig. 6).

## Discussion

In the present in vitro study, we evaluated the inflammatory and osteogenic response induced by Ti6Al4V particles released during implantoplasty and by as-received commercially pure Ti particles. To the best of our knowledge, this is the first paper to analyze the immunological effects induced by Ti6Al4V debris released during IP upon THP-1 and BM-MSCs. Our results suggests that both commercially pure Ti and Ti6Al4V particles may trigger an inflammatory response, because they can promote the expression and release of proinflammatory genes and cytokines. Moreover, Ti6Al4V extracts produced a significant decrease in the osteogenic markers Runx2 and OC in comparison with control osteogenic medium, but there was no effect on ALP activity.

Several limitations of the present study should be disclosed. Firstly, although this study followed ISO 10993-5 for the performance of cellular assays, due to the biological nature and complexity of the process of inflammation, future studies should assess the immunological response triggered by metal particles released during IP in an in vivo experimental scenario. Secondly, THP-1 cell line has limitations because does not consistently behave as human macrophages^33^. However, it must be pointed out that the genetic homogeneity of THP-1 minimizes the degree of variability in the cell phenotype, which facilitates reproducibility of findings^33,34^. Finally, as grinding changes the properties of the metal and renders it more susceptible to corrosion^32^, using IP debris from commercially pure Ti implants could be interesting.

Titanium is a transitional element used in dental implantology and orthopedics due to its high biocompatibility, corrosion resistance and mechanical properties^35^. The vast majority of osseointegrated dental implants are made of c.p. Ti or Ti alloys^36^. Ti6Al4V alloy contains 6% of Al and 4% of V which differs from the composition of c.p. Ti^36^. Both materials are considered bioinert due to the formation of a spontaneous oxide layer composed mainly of TiO_2_ but which may incorporate other small contents of Al or V^37^. This protective oxide layer can be lost by corrosion^38^, generating ions and nanoparticles that can remain in the peri-implant tissues or migrate systemically^19,39^. Regarding Ti6Al4V alloys, a previous in vitro study showed that the metal particles of implantoplasty release higher concentration of V and Al ions compared to Ti ions^31^. Both ions have been associated with local adverse effects, neurotoxicity and negative cell viability response^40–42^. In the present study, we used a dilution 1:100 because it allowed the viability of the used cells. To the best of authors’ knowledge, there are no clinical studies in the literature describing the concentration of metal ions in the peri-implant tissues after implantoplasty^43^. Therefore, future in vivo studies are needed to evaluate the effect of Ti, Al and V ion concentrations on peri-implant tissues.

Some concerns have been raised during the past few years regarding the role of Ti and metal particles in the development of aseptic osteolysis or “peri-implantitis” in dental implants^1,26^. Therefore, we decided to conduct an in vitro analysis of the inflammatory and osteogenic response triggered by the metal debris released during IP. Firstly, we determined the cytotoxicity of each extract of Ti particles (Ti6Al4V and commercially pure Ti) in the THP-1 macrophage cell line at 24 h and 48 h. Titanium extracts reduced THP-1 cell viability at concentrations higher than 1:10 dilution, while in the case of Ti6Al4V extracts this phenomenon occurred at concentrations higher than 1:100 dilution (Fig. 1). This finding is consistent with the data from previous publications reporting that the release of Al and V ionic species may have a detrimental effect upon cells^40,42,44^. On the other hand, both extracts (Ti6Al4V and commercially pure Ti) induced a proinflammatory response by promoting the polarization of macrophages towards M1 through the expression and release of proinflammatory genes and cytokines. We found some discrepancies between levels of mRNA and protein, which is a common finding in this type of cell culture assay^45,46^. Eger et al.^30^ reported that metal particles detached during the scaling of SLA dental implants were engulfed by macrophages and triggered a marked inflammatory response, with the release of IL-1β, IL-6, TNF-α. These cytokines are also related to the activation of osteoclasts via the activation of RANKL expression, thereby inducing bone resorption^17,37,47^.

Regarding osteogenic response, Ti extracts reduced the viability of the cultured BM-MSCs at concentrations > 1:10 dilution, while in the case of Ti6Al4V extracts this occurred at concentrations > 1:100 dilution (Fig. 4). Titanium particles can affect BM-MSC viability by inducing apoptosis through activation of the tumour suppressor proteins p53 and p73—this circumstance being dependent upon material composition, particle dosage and time^48^. Once cytotoxicity was evaluated, we analysed gene expression and ALP activity of the BM-MSCs. Gene expression assays showed that the Ti6Al4V alloy could inhibit osteogenesis—its effects being particularly relevant in relation to the Runx2 and OC markers (Fig. 5), which are an early and late indicator, respectively, of osteogenic differentiation^49,50^. On the other hand, we found no relevant differences regarding the activity of ALP (Fig. 6), an enzyme that releases inorganic phosphate, needed for the mineralization of bone tissue.

Some controversy has arisen regarding the use of antibiotic in in vitro cell culture studies^51^. The use of antibiotic can affect different aspects of the cells, such as cell metabolism or cell differentiation^52,53^. In this line, Ryu et al.^51^ highlighted that penicillin–streptomycin antibiotic can alter gene expression in a human cell line. In contrast, antibiotics use has been recommended by standard cell culture protocols to maintain aseptic conditions and to reduce the risk of sample contamination^53^. This fact is especially important in THP-1 cell line since these cells are sensible to bacteria and their metabolic products^54^. Thus, we decided to use 1% penicillin–streptomycin in both cell culture to reduce bacterial contamination that could impact on macrophage polarization. Future studies should analyse the influence of not introducing an antibiotic in cell assays with metal particles.

In a way similar to the dental clinical scenario, metal particles are also generated in orthopedics, though in this case through friction of the joint surfaces. Such released metal debris can enter the cells via endocytosis and induce adverse biological effects, such as aseptic osteolysis^55–58^. Obando-Pereda et al.^59^ reported that these wear-generated particles triggered the expression of proinflammatory cytokines such as TNF-α, IL-1β and IL-6. The release of these cytokines may also induce osteoclastogenesis and lead to aseptic loss of the joint prosthesis^60,61^. We found that both Ti6Al4V particles generated by IP and commercially pure Ti particles activated the abovementioned cytokines. Furthermore, Ti6Al4V particles decreased the expression of Runx2 and OC, which means that such metal debris may inhibit osteogenesis. Indeed, Runx2 plays a pivotal role in osteoblast differentiation and in bone formation through transcriptional regulation of their target genes, while OC is involved in osteoblast activity and mineralization in the later stages of bone formation^62^.

## Conclusions

Within the limitations of the present study, Ti6Al4V and c.p. Ti metal particles increased proinflammatory genes and cytokines. In turn, Ti6Al4V particles generated by implantoplasty reduced the expression of osteogenic markers. Animal and human studies are needed in order to confirm the possible role of Ti6Al4V and Ti particles in the development of bone resorption or peri-implant inflammation.

## Methods

### Titanium metal particles

In the present in-vitro study, use was made of a previously published protocol to collect metal particles released during IP of Ti6Al4V dental implants^31,32^. In order to replicate the clinical procedure of IP, metal particles were collected from Ti grade V dental implants (Ti6Al4V) using a standardized procedure consisting of the sequential use of a ﻿fine-grained tungsten carbide bur and two silicon carbide polishers^63^. The characterization of the material sample, including composition, granulometry, crystalline structure, morphology, ion release, nanoindentation and corrosion behaviour has been described in previous publications^31,32^. On the other hand, a Ti powder, purchased from NanoShel (Chapel St., UK), was used as control in order to compare with inflammatory and osteogenic cell cultures with the tested Ti alloy IP particles (Ti6Al4V). This c.p. Ti has a powder size distribution that ranged from 30 to 70 nm, which was similar to the size of some metal particles released during implantoplasty^31^.

### Sterilization of samples of Ti and collection of extracts

We separately sterilized IP debris (test) and c.p. Ti powder (control) with 96° ethanol. The latter was eliminated by three centrifugation cycles at 7200 rpm during 5 min and washing with Dulbecco’s Phosphate Buffered Saline (DPBS) (Sigma-Aldrich, Sant Louis, USA). After the last centrifugation, DPBS was discarded, and the required volume of cell culture medium was added in order to obtain a final concentration of 0.2 g of sample per ml of medium. Cell assays were performed by indirect culture with extracts according to section 8.2 of ISO 10993–5: after separation of metal particles and medium, we carried out the assays using this culture medium that had been previously exposed to metal particles.

Both samples (Ti6Al4V and c.p. Ti) were incubated for 72 h at 37 °C. In the case of the assays performed with the THP-1 macrophage cell line, the metal particles were incubated in supplemented Roswell Park Memorial Institute (RPMI) 1640 medium with L-glutamine and sodium bicarbonate (reference: r8758, Sigma-Aldrich, Sant Louis, USA) and supplemented with 10% fetal bovine serum (FBS, Sigma-Aldrich, Sant Louis, USA) and 1% penicillin–streptomycin (Fisher Scientific, Hampton, USA). On the other hand, in the assays with BM-MSCs we used the mesenchymal stem cell basal medium (reference: C-28013, Sigma-Aldrich, Sant Louis, USA) supplemented with 125 pg/ml rh FGF basic, 15 ng/ml rh IGF-1, 7% FBS, 2.4 mM L-Alanyl-L-Glutamine (reference: PCS-500–041, ATCC, Manassas, USA) and 1% penicillin–streptomycin (Fisher Scientific, Hampton, USA).

### Cell culture and differentiation of THP-1 macrophage cell line

The THP-1 macrophage cell line was obtained from DSMZ (ACC 16) (Braunschweig, Germany) and the cell suspension culture was maintained at 3 × 10^5^ cells/ml in RPMI 1640 medium (Sigma-Aldrich, Sant Louis, USA) supplemented with 10% fetal bovine serum (FBS) (Sigma-Aldrich, Sant Louis, USA) and 1% penicillin–streptomycin (Fisher Scientific, Hampton, USA). Cells were cultured in a humidity-controlled incubator at 37 °C with 5% CO_2_ supply. To enhance adhesion of the cells to the culture plates, we seeded them at a density of 3 × 10^4^ cells/cm^2^ in the presence of phorbol 12-myristate 13-acetate (PMA) (Sigma-Aldrich, Sant Louis, USA) for 6 h. Then, the medium with PMA was removed, washed with DPBS, and the cells were exposed to the extracts previously obtained. In all assays, cells cultured with medium containing a concentration of 100 ng/ml lipopolysaccharide (LPS) (Sigma-Aldrich, Sant Louis, USA) were used as a positive control for inflammation^64^, and cells cultured without extract (tissue culture plate, TCP) were used as negative controls for inflammation.

### Analysis of cytotoxicity in macrophage cell culture

An indirect contact cytotoxicity test was performed according to the guidelines specified in UNE EN ISO 10,993-5: "Biological evaluation of medical devices" part 5, entitled: "In vitro cytotoxicity tests". Cytotoxicity was calculated with the cell survival index, which indicates cytotoxicity at < 80%.

Cells were incubated for 24 and 48 h with different dilutions of the extract: undiluted and diluted extract 1:2, 1:10, 1:100 and 1:1000, using complete medium for the dilutions. We assessed cell adhesion and morphology by light microscopy before and after contact with the extracts. Once the assay was completed, we assessed cell viability through metabolic activity using resazurin sodium salt reagent (Sigma-Aldrich, Sant Louis, USA). Accordingly, we cultured cells in their medium with resazurin (10 μg/ml) for 3 h in the incubator at 37 °C and 5% CO_2_. Then, we measured absorbance at 570 and 600 nm using a plate reader (Infinite 200 PRO, Tecan, Männedorf, Switzerland).

### Macrophage gene expression analysis

Gene expression was analyzed by real-time quantitative polymerase chain reaction (RT-qPCR). We analyzed proinflammatory markers (CCR7, TNF-α and IL-1β genes) and anti-inflammatory markers (CD206, TGF-β and IL-10 genes). In turn, RNA was isolated according to the recommendations of the manufacturer with the NucleoSpin RNA kit (Macherey–Nagel, Düren, Germany), which included DNase treatment. Once extracted, we quantified RNA using a microplate reader (Take3, Bio-Tek, Winooski, USA). Then, we retrotranscribed RNA into cDNA using the Transcriptor First Strand cDNA Synthesis kit (Roche, Basel, Switzerland) according to the recommendations of the manufacturer. We used qPCR to detect gene expression with the QuantiNova SYBR Green PCR Kit (Qiagen, Hilden, Germany): 10 ng of cDNA was amplified by an initial activation step of two minutes at 95 °C, denaturation for 5 s at 95 °C, and 40 cycles of hybridization/extension for 10 s at 60 °C using the CFX96 Real-Time System (BioRad, Hercules, USA). Primers used to detect specific genes in the amplification are detailed in Table 1. We normalized gene expression to the mean cycle threshold (Ct) of the constitutive β-actin gene. The 2^−ΔΔ^Ct method was used to compare mRNA expression between conditions, taking the cells cultured in tissue culture plate (TCP) value as reference.

**Table 1:** List of primer sequences used for the gene expression analysis.

		Gene	Forward (sequence 5’-3’)	Reverse (sequence 5’-3’)
Inflammation-associated genes	M1 (pro inflammatory)	TNF-α	TTCCAGACTTCCTTGAGACACG	AAACATGTCTGAGCCAAGGC
		IL-1β	GACACATGGGATAACGAGGC	ACGCAGGACAGGTACAGATT
		CCR7	GGCTGGTCGTGTTGACCTAT	ACGTAGCGGTCAATGCTGAT
	M2 (anti-inflammatory)	IL-10	AAGCCTGACCACGCTTTCTA	ATGAAGTGGTTGGGGAATGA
		TGF-β	TTGATGTCACCGGAGTTGTG	TGATGTCCACTTGCAGTGTG
		CD206	CCTGGAAAAAGCTGTGTGTCAC	AGTGGTGTTGCCCTTTTTGC
Osteogenesis-associated genes		Runx2	CCCGTGGCCTTCAAGGT	CGTTACCCGCCATGACAGTA
		ALP	GGAACTCCTGACCCTTGACC	TCCTGTTCAGCTCGTACTGC
		OC	CGCCTGGGTCTCTTCACTAC	CTCACACTCCTCGCCCTATT
Housekeeping gene		β-actin	AGAGCTACGAGCTGCCTGAC	AGCACTGTGTTGGCGTACAG

### Macrophage cytokine release analysis

After culturing the macrophages with the extracts for 24 and 48 h, we collected the supernatants to quantify the release of proinflammatory (TNF-α and IL-1β) and anti-inflammatory (IL-10) cytokines by macrophages into the culture medium. Quantification was performed using commercially available ELISA kits (Thermofisher Scientific, Waltham, USA), following the recommendations of the manufacturer.

### Cell culture and differentiation of in human bone marrow-derived mesenchymal stem cells (BM-MSCs)

The BM-MSCs were obtained from ATCC (PCS-500-012) and cultured in Mesenchymal Stem Cell Basal Medium (ATCC; PCS-500-030) supplemented with 125 pg/ml rhFGF basic, 15 ng/ml rhIGF-1, 7% FBS, 2.4 mM L-alanyl-L-glutamine (ATCC; PCS-500-041) and 1% penicillin–streptomycin (Fisher Scientific, Hampton, USA). We kept the cell culture density at 3000 cells/cm^2^. Subsequently, for cytotoxicity assay, BM-MSCs were seeded at a density of 9500 cells/cm^2^, while for gene expression, alkaline phosphatase (ALP) activity and proliferation assays we seeded them at a density of 8000 cells/cm^2^. In all assays, extracts were generated using osteogenic medium, which was composed of medium supplemented with 10 mM β-glycerophosphate, 50 μg/ml ascorbic acid and 0.1 μM dexamethasone (Sigma-Aldrich, Sant Louis, USA). As control, we used BM-MSCs exposed to osteogenic medium but without contact with the microparticles.

### Analysis of cytotoxicity BM-MSCs culture

The BM-MSCs were exposed for 3 and 7 days to different dilutions of the extracts made using osteogenic medium: undiluted and diluted extract 1:2, 1:10, 1:100 and 1:1000. Metabolic activity was then analyzed by resazurin reduction assay. The BM-MSCs were incubated for 45 min at 37 °C in the presence of medium with a concentration of 50 μg/ml of resazurin sodium salt. Resazurin reduction was quantified in the same manner as described in previous sections.

### BM-MSCs gene expression analysis

Gene expression was analyzed at days 3, 7, 14 and 21 by RT-qPCR using the same protocol as that employed for the THP-1 gene expression assays. The genes analyzed were Runx2, alkaline phosphatase (ALP) and osteocalcin (OC). Primers used to amplify these genes are described in Table 1.

### Alkaline phosphatase (ALP) activity assay

To quantify the ALP activity in the samples, we used a colorimetric method based on the conversion of p-nitrophenyl phosphate to p-nitrophenol in the presence of ALP. We lysed samples cultured with the extracts and with osteogenic medium with 200 μl of 0.1% Triton X-100 in 1xTE buffer (Sigma-Aldrich, Sant Louis, USA), followed by three freeze–thaw cycles. Subsequently, 50 μl of sample were combined with 50 μl of a mixture in 1:1:1 ratios of 1.5 M 2-amino-2-methyl-1-propanol buffer (Sigma-Aldrich, Sant Louis, USA), 20 mM phosphatase substrate solution (Sigma-Aldrich, Sant Louis, USA) and 1 mM MgCl_2_. The samples were incubated for 30 min at 37 °C, and we stopped the reaction using 1 M NaOH. The production of p-nitrophenol was quantified by measuring the absorbance at 405 nm and comparing it against a standard curve prepared with known concentrations of p-nitrophenol. Results were then normalized to the results obtained in the proliferation assay. Cell proliferation was quantified by measuring the amount of dsDNA in the samples with the Quant-iT PicoGreen dsDNA Assay Kit (Invitrogen, Waltham, USA) following the recommendations of the manufacturer. We mixed 100 μl of sample and 100 μl of a 1:200 dilution of PicoGreen reagent in a black 96-well plate.

### Statistical analysis

All assays were performed in triplicate, except for ALP activity, which was conducted in quadruplicate. Data were expressed as the mean ± standard deviation (SD). Statistical analysis was performed using MINITAB (version 18, Minitab Inc.). Nonparametric testing was used, for although the normal distribution of each dataset was confirmed by the Anderson–Darling normality test, homoscedasticity was ruled out (Barlett and Levene's test for homogeneity of variances). Therefore, we used the Kruskal–Wallis test for multiple comparisons and the Mann–Whitney U-test for individual (one-to-one) comparisons. Statistical significance was considered for *p* < 0.05*.*

## Data Availability

The datasets generated and/or analysed during the current study are available in the Gene Expression Omnibus (GEO) repository, https://www.ncbi.nlm.nih.gov/geo/query/acc.cgi?acc=GSE202419.

## References

[1] Schwarz F, Derks J, Monje A, Wang HL (2018). Peri-implantitis. J. Clin. Periodontol..

[2] Schlee M (2021). Treatment of periimplantitis with electrolytic cleaning versus mechanical and electrolytic cleaning: 18-month results from a randomized controlled clinical trial. J. Clin. Med..

[3] Suarez F, Monje A, Galindo-Moreno P, Wang HL (2013). Implant surface detoxification: A comprehensive review. Implant Dent..

[4] Figuero E, Graziani F, Sanz I, Herrera D, Sanz M (2014). Management of peri-implant mucositis and peri-implantitis. Periodontol..

[5] Zipprich H (2022). Comparison of decontamination efficacy of two electrolyte cleaning methods to diode laser, plasma, and air - abrasive devices. Clin. Oral Investig..

[6] Gosau M (2010). Effect of six different peri-implantitis disinfection methods on in vivo human oral biofilm. Clin. Oral Implants Res..

[7] Pranno N (2021). Comparison of the effects of air-powder abrasion, chemical decontamination, or their combination in open-flap surface decontamination of implants failed for peri-implantitis : An ex vivo study. Clin. Oral Investig..

[8] Kolonidis SG (2003). Osseointegration on implant surfaces previously contaminated with plaque. An experimental study in the dog. Clin. Oral Implants Res..

[9] Alhag M, Renvert S, Polyzois I, Claffey N (2008). Re-osseointegration on rough implant surfaces previously coated with bacterial biofilm: An experimental study in the dog. Clin. Oral Implants Res..

[10] Schwarz F, Sahm N, Iglhaut G, Becker J (2011). Impact of the method of surface debridement and decontamination on the clinical outcome following combined surgical therapy of peri-implantitis: a randomized controlled clinical study. J. Clin. Periodontol..

[11] Schwarz F, John G, Mainusch S, Sahm N, Becker J (2012). Combined surgical therapy of peri-implantitis evaluating two methods of surface debridement and decontamination. A two-year clinical follow up report. J. Clin. Periodontol..

[12] Schwarz F (2017). Combined surgical therapy of advanced peri-implantitis evaluating two methods of surface decontamination: A 7-year follow-up observation. J. Clin. Periodontol..

[13] Berglundh T (2018). Peri-implant diseases and conditions: Consensus report of workgroup 4 of the 2017 world workshop on the classification of periodontal and peri-implant diseases and conditions. J. Clin. Periodontol..

[14] Heitz-Mayfield LJA, Salvi GE (2018). Peri-implant mucositis. J. Periodontol..

[15] Cadosch D (2010). Biocorrosion and uptake of titanium by human osteoclasts. J. Biomed. Mater. Res. Part A.

[16] Fretwurst T (2018). Is metal particle release associated with peri-implant bone destruction ? An Emerging concept. J. Dent. Res..

[17] Pettersson M (2017). Titanium ions form particles that activate and execute interleukin-1β release from lipopolysaccharide-primed macrophages. J. Periodontal Res..

[18] Suárez-López del Amo F, Garaicoa-Pazmiño C, Fretwurst T, Castilho RM, Squarize CH (2018). Dental implants-associated release of titanium particles: A systematic review. Clin. Oral Implants Res..

[19] Olmedo DG, Nalli G, Verdú S, Paparella ML, Cabrini RL (2013). Exfoliative cytology and titanium dental implants: A pilot study. J. Periodontol..

[20] Suárez-López del Amo F (2017). Titanium activates the dna damage response pathway in oral epithelial cells: A pilot study. Int. J. Oral Maxillofac. Implants.

[21] Insua A, Monje A, Wang HL, Miron RJ (2017). Basis of bone metabolism around dental implants during osseointegration and peri-implant bone loss. J. Biomed. Mater. Res. Part A.

[22] Garlet GP, Giannobile WV (2018). Macrophages: The bridge between inflammation resolution and tissue repair?. J. Dent. Res..

[23] Wang X, Li Y, Feng Y, Cheng H, Li D (2019). Macrophage polarization in aseptic bone resorption around dental implants induced by Ti particles in a murine model. J. Periodontal Res..

[24] Pajarinen J (2013). The response of macrophages to titanium particles is determined by macrophage polarization. Acta Biomater..

[25] Mandelin J (2003). Imbalance of RANKL/RANK/OPG system in interface tissue in loosening of total hip replacement. J. Bone Jt. Surg. - Ser. B.

[26] Albrektsson T, Canullo L, Cochran D, De Bruyn H (2016). “Peri-implantitis”: A complication of a foreign body or a man-made “disease”. Facts and fiction. Clin. Implant Dent. Relat. Res..

[27] Mombelli A, Hashim D, Cionca N (2018). What is the impact of titanium particles and biocorrosion on implant survival and complications? A critical review. Clin. Oral Implants Res..

[28] Kheder W, Al Kawas S, Khalaf K, Samsudin AR (2021). Impact of tribocorrosion and titanium particles release on dental implant complications — A narrative review. Jpn. Dent. Sci. Rev..

[29] Noumbissi S, Scarano A, Gupta S (2019). A literature review study on atomic ions dissolution of titanium and its alloys in implant dentistry. Materials (Basel)..

[30] Eger M, Sterer N, Liron T, Kohavi D, Gabet Y (2017). Scaling of titanium implants entrains inflammation-induced osteolysis. Sci. Rep..

[31] Toledano-Serrabona J (2021). Physicochemical and biological characterization of Ti6Al4V particles obtained by implantoplasty: An in vitro study. Part I. Materials (Basel).

[32] Toledano-Serrabona J (2021). Mechanical properties and corrosion behavior of Ti6Al4V particles obtained by implantoplasty: An in vitro study. Part II. Materials (Basel).

[33] Chanput W, Mes JJ, Wichers HJ (2014). THP-1 cell line: An in vitro cell model for immune modulation approach. Int. Immunopharmacol..

[34] Bosshart H, Heinzelmann M (2016). THP-1 cells as a model for human monocytes. Ann. Transl. Med..

[35] Nicholson W (2020). Titanium alloys for dental implants: A review. Prosthesis.

[36] Elias CN, Fernandes DJ, De Souza FM, Monteiro EDS, De Biasi RS (2019). Mechanical and clinical properties of titanium and titanium-based alloys (Ti G2, Ti G4 cold worked nanostructured and Ti G5) for biomedical applications. J. Mater. Res. Technol..

[37] Messous R (2021). Cytotoxic effects of submicron- and nano-scale titanium debris released from dental implants: An integrative review. Clin. Oral Investig..

[38] Wilson TG (2021). Bone loss around implants—is it metallosis?. J. Periodontol..

[39] Toledano-Serrabona, J. *et al.* Ion release and local effects of titanium metal particles from dental implants. An experimental study in rats. *J. Periodontol.* Epub ahead of print (2022) 10.1002/JPER.22-0091 PMC10087269 35678251

[40] Willis J, Crean SJ, Barrak FN (2021). Is titanium alloy Ti-6Al-4V cytotoxic to gingival fibroblasts — A systematic review. Clin. Exp. Dent. Res..

[41] Cordeiro JM, Barão VAR (2017). Is there scienti fi c evidence favoring the substitution of commercially pure titanium with titanium alloys for the manufacture of dental implants ?. Mater. Sci. Eng. C.

[42] Zwolak I (2014). Vanadium carcinogenic, immunotoxic and neurotoxic effects: A review of in vitro studies. Toxicol. Mech. Methods.

[43] Noronha Oliveira M (2018). Can degradation products released from dental implants affect peri-implant tissues?. J. Periodontal Res..

[44] Challa VSA, Misra RDK (2013). Reduced toxicity and superior cellular response of preosteoblasts to Ti-6Al-7Nb alloy and comparison with Ti-6Al-4V. J. Biomed. Mater. Res. A..

[45] Dodo CG (2017). Pro-inflammatory analysis of macrophages in contact with titanium particles and porphyromonas gingivalis. Braz. Dent. J..

[46] Koussounadis A, Langdon SP, Um IH, Harrison DJ, Smith VA (2015). Relationship between differentially expressed mRNA and mRNA-protein correlations in a xenograft model system. Sci. Rep..

[47] Eger M (2018). Mechanism and prevention of titanium particle-induced inflammation and osteolysis. Front. Immunol..

[48] Wang ML (2003). Direct and indirect induction of apoptosis in human mesenchymal stem cells in response to titanium particles. J. Orthop. Res..

[49] Gromolak S (2020). Biological characteristics and osteogenic differentiation of ovine bone marrow derived mesenchymal stem cells stimulated with FGF-2 and BMP-2. Int. J. Mol. Sci..

[50] Loebel C (2015). In vitro osteogenic potential of human mesenchymal stem cells is predicted by Runx2/Sox9 ratio. Tissue Eng. - Part A.

[51] Ryu AH, Eckalbar WL, Kreimer A, Yosef N, Ahituv N (2017). Use antibiotics in cell culture with caution: Genome-wide identification of antibiotic-induced changes in gene expression and regulation. Sci. Rep..

[52] Smith RP (2014). Genome-wide discovery of drug-dependent human liver regulatory elements. PLoS Genet..

[53] Llobet L, Montoya J, López-Gallardo E, Ruiz-Pesini E (2015). Side effects of culture media antibiotics on cell differentiation. Tissue Eng. Part C. Methods.

[54] Vargas-Hernández O (2020). THP-1 cells increase TNF-α production upon LPS + soluble human IgG co-stimulation supporting evidence for TLR4 and Fcγ receptors crosstalk. Cell. Immunol..

[55] Gargioli C (2018). Oxidative stress preconditioning of mouse perivascular myogenic progenitors selects a subpopulation of cells with a distinct survival advantage in vitro and in vivo. Cell Death Dis..

[56] Yao JJ (2017). Local cellular responses to titanium dioxide from orthopedic implants. Biores. Open Access.

[57] Haleem-Smith H (2012). Biological responses of human mesenchymal stem cells to titanium wear debris particles. J. Orthop. Res..

[58] Okafor CC, Haleem-Smith H, Laqueriere P, Manner PA, Tuan RS (2006). Particulate endocytosis mediates biological responses of human mesenchymal stem cells to titanium wear debris. J. Orthop. Res..

[59] Obando-Pereda GA, Fischer L, Stach-Machado DR (2014). Titanium and zirconia particle-induced pro-inflammatory gene expression in cultured macrophages and osteolysis, inflammatory hyperalgesia and edema in vivo. Life Sci..

[60] Masui T, Sakano S, Hasegawa Y, Warashina H, Ishiguro N (2005). Expression of inflammatory cytokines, RANKL and OPG induced by titanium, cobalt-chromium and polyethylene particles. Biomaterials.

[61] Xu J (2009). NF-κB modulators in osteolytic bone diseases. Cytokine Growth Factor Rev..

[62] Makita N (2008). Two of four alternatively spliced isoforms of RUNX2 control osteocalcin gene expression in human osteoblast cells. Gene.

[63] Costa-Berenguer X (2018). Effect of implantoplasty on fracture resistance and surface roughness of standard diameter dental implants. Clin. Oral Implants Res..

[64] Díez-Tercero L, Delgado LM, Bosch-Rué E, Perez RA (2021). Evaluation of the immunomodulatory effects of cobalt, copper and magnesium ions in a pro inflammatory environment. Sci. Rep..

